# APE1 polymorphic variants cause persistent genomic stress and affect cancer cell proliferation

**DOI:** 10.18632/oncotarget.8477

**Published:** 2016-03-30

**Authors:** Lisa Lirussi, Giulia Antoniali, Chiara D'Ambrosio, Andrea Scaloni, Hilde Nilsen, Gianluca Tell

**Affiliations:** ^1^ Laboratory of Molecular Biology and DNA Repair, Department of Medical and Biological Sciences, University of Udine, 33100 Udine, Italy; ^2^ Proteomics and Mass Spectrometry Laboratory, ISPAAM, National Research Council, 80147 Naples, Italy; ^3^ Department of Clinical Molecular Biology, University of Oslo and Akershus University Hospital, Nordbyhagen 1474, Norway

**Keywords:** APE1/Ref-1, genotoxic damage, genetic variants, replication stress

## Abstract

Apurinic/apyrimidinic endonuclease 1 (APE1) is the main mammalian AP-endonuclease responsible for the repair of endogenous DNA damage through the base excision repair (BER) pathway. Molecular epidemiological studies have identified several genetic variants associated with human diseases, but a well-defined functional connection between mutations in APE1 and disease development is lacking. In order to understand the biological consequences of APE1 genetic mutations, we examined the molecular and cellular consequences of the selective expression of four non-synonymous APE1 variants (L104R, R237C, D148E and D283G) in human cells. We found that D283G, L104R and R237C variants have reduced endonuclease activity and impaired ability to associate with XRCC1 and DNA polymerase β, which are enzymes acting downstream of APE1 in the BER pathway. Complementation experiments performed in cells, where endogenous APE1 had been silenced by shRNA, showed that the expression of these variants resulted in increased phosphorylation of histone H2Ax and augmented levels of poly(ADP-ribosyl)ated (PAR) proteins. Persistent activation of DNA damage response markers was accompanied by growth defects likely due to combined apoptotic and autophagic processes. These phenotypes were observed in the absence of exogenous stressors, suggesting that chronic replication stress elicited by the BER defect may lead to a chronic activation of the DNA damage response. Hence, our data reinforce the concept that non-synonymous APE1 variants present in the human population may act as cancer susceptibility alleles.

## INTRODUCTION

Genomic integrity is constantly challenged by the action of exogenous and endogenous DNA damaging agents, such as ionizing radiation and reactive oxygen species generated by mitochondrial metabolism [1]. A link between loss of DNA repair function and carcinogenesis has been documented [2], and several epidemiological studies have implicated single nucleotide polymorphisms (SNPs) in DNA repair genes in cancer predisposition [3–5]. For instance, individual SNPs in Base Excision Repair (BER) proteins have been genetically correlated with colorectal, breast and lung cancer predisposition [5]. Moreover, it is likely that disease phenotypes may derive from a combination of variations in functionally coupled proteins, as in the case of DNA polymerase β (Polβ), X-ray repair cross-complementing protein 1 (XRCC1) and apurinic/apyrimidinic endonuclease 1 (APE1), which would affect the capacity to repair DNA damage through the BER pathway [5–7]. Most polymorphisms in genes encoding BER proteins are low penetrance susceptibility alleles and a clear demonstration of the functional consequences *in vivo* of these polymorphisms is still missing.

APE1 is an essential enzyme that has a coordinating function in the BER pathway. It processes AP-sites generated by DNA glycosylases that remove damaged bases as the first step of BER. Loss of APE1 function will therefore lead to an accumulation of DNA repair intermediates that are both mutagenic and cytotoxic. Several non-synonymous APE1 genetic variants, *e.g.* L104R, R237C, D148E and D283G, have been identified in the human population [5]. Among these APE1 missense variants, D148E is the most frequent SNP in the normal population [8]. L104R and D283G have been uniquely associated with amyotrophic lateral sclerosis (ALS), although the validity of these variants is a matter of debate [8, 9]. R237C is a variant associated with endometrial cancer [8, 10] (Table 1). With the exception of mutations at the catalytic residue D283, none of these substitutions occurs at residues responsible for known APE1 functions. It has been proposed that this may be related to a strong negative selection pressure elicited by the essential functions of APE1. However, no data are available to support this hypothesis at the molecular level. Interestingly, some polymorphisms occur in the N-terminal domain of APE1, a region harboring a number of residues that are subjected to post-translational modifications (PTMs) and are essential for a proper interaction with other proteins. This observation suggests that APE1 SNPs may indirectly impact on protein function by affecting its regulation or its ability to interact with specific binding partners [11–14]. To date, various studies have characterized the *in vitro* endonuclease and exonuclease activity of APE1 mutants using recombinant proteins expressed in *E. coli* [8, 9, 15]. However, these studies were not designed to capture indirect consequences of amino acid substitutions that do not affect catalytic properties. Hence, a systematic characterization of the functional consequences of the expression of APE1 genetic variants is still missing.

**Table 1:** Predicted impact of APE1 variants on protein structure/function

APE1genetic variants	Source	Percentageoffrequency	Functionalconsequences	PROVEAN		SIFT		CUPSAT		PolyPhen-2	
				Score	Prediction	Score	Prediction	Overallstability	Torsion	Score	Prediction
L104R	ALS	once	Reduced AP endonuclease activity (~40%)[9].Impaired 3′-RNA phosphatase and endoribonuclease activities [15, 20]	−4.925	Deleterious	0.00	Affectedproteinfunction	Stabilizing	Unfavorable	0.987	Probably damaging
D148E	NCBI rs1130408	48.5	Normal AP endonuclease activity [8, 9]. Impaired 3′-RNA phosphatase and endoribonuclease activities [15, 20]	−0.204	Neutral	1.00	Tolerated	Destabilizing	Favorable	0.000	Benign
R237C	Tumor, NCBI rs375526265	once	Reduced 3′ to5′ exonuclease and 3′-damage excision activities; slightly reduced AP-DNA complex stability [8].Reduced incision capacity in proximity of nucleosomes [21]	−7.884	Deleterious	0.00	Affected protein function	Stabilizing	Unfavorable	1.000	Probably damaging
D283G	ALS	once	Reduced AP endonuclease activity (~90%) [9]	−6.910	Deleterious	0.00	Affected protein function	Destabilizing	Unfavorable	1.000	Probably damaging

For PROVEAN software (http://provean.jcvi.org/index.php), a cut-off score ≤ −2.5 is considered as deleterious, while a value greater than that is predicted as neutral. For SIFT modelling software (http://sift.jcvi.org/), a score ≤ 0.05 is considered as deleterious, whereas > 0.05 is predicted as tolerated. As template, the APE1 sequence (NP_001632.2) was used. For the PolyPhen-2 tool (http://genetics.bwh.harvard.edu/pph2/) and PROVEAN model, APE1 UniProtKB sequence (P27695) was used as query. CUPSAT predictions (http://cupsat.tu-bs.de/) were obtained by using the PDB APE1 crystallographic structure (1DE8) and the thermal experimental method. N/A = not available. Once = observed a single time. APE1 sequence entries reported above for computational analyses were identical and corresponded to the wild-type enzyme.

For a better understanding of the correlation between APE1 polymorphisms and susceptibility for disease, we characterized the impact of expressing L104R, R237C, D148E and D283G APE1 variants in HeLa cells where endogenous wild-type APE1 was silenced by shRNA. Here, we present data demonstrating that these variants severely impact on protein ability of binding to BER enzymes XRCC1 and Polβ. Expression of these APE1 genetic variants led to a persistent activation of the DNA damage response in the absence of exogenous DNA damaging agents, thus reinforcing the concept that APE1 variants may act as cancer susceptibility alleles.

## RESULTS

### Computational evaluation of the effect of some polymorphic variants on APE1 structure and function

**Figure 1:**
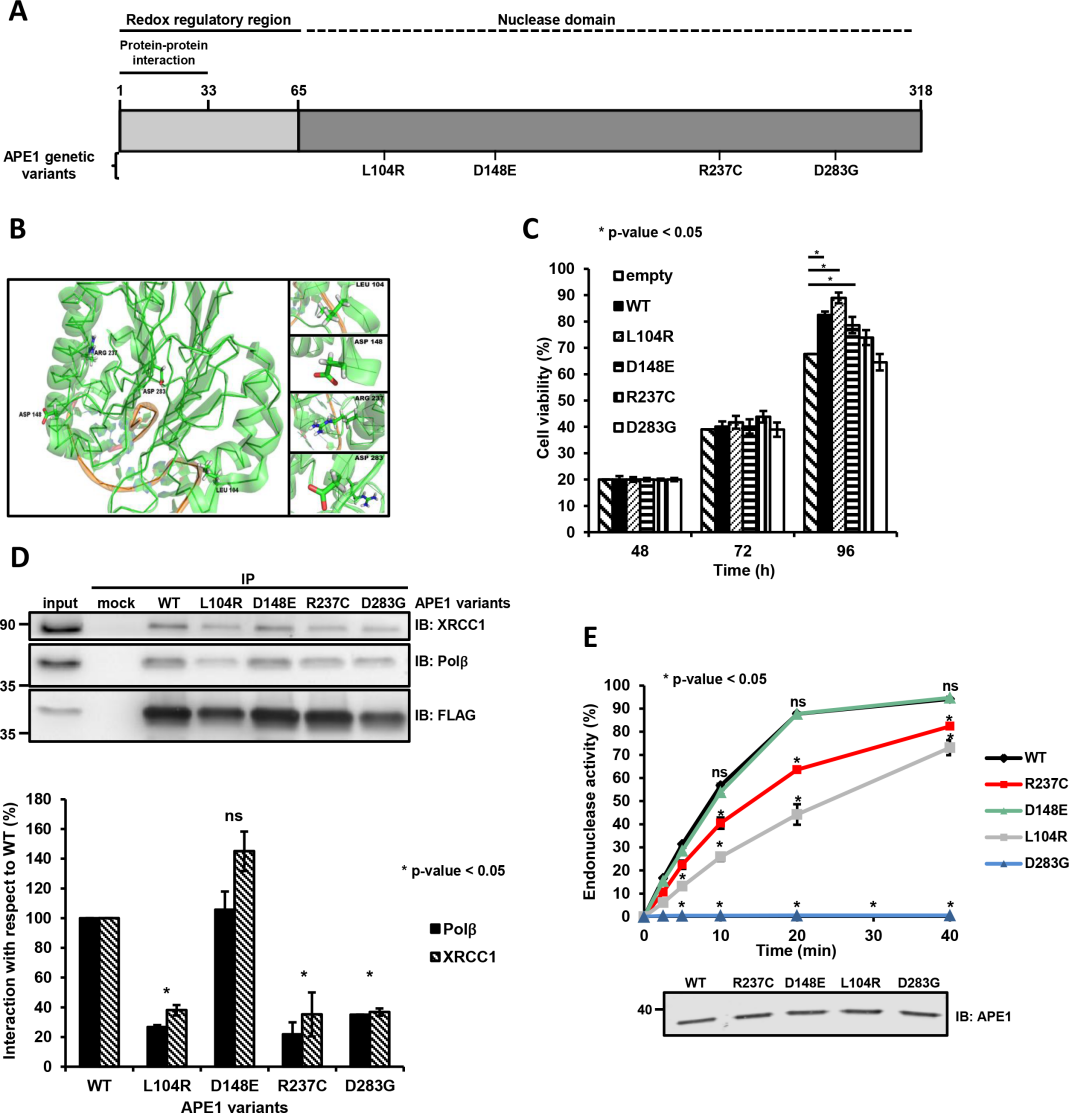
Substitution mutations in APE1 affects protein function (**A** and **B**) APE1 amino acid substitutions and corresponding localization within the APE1 structure. Position of the APE1 mutations in a linear sequence (A) and three-dimensional protein representation (B). The first 33 residues involved in protein-protein interaction, the redox regulatory region and the nuclease domain are shown (A). The coordinates of the protein structure were retrieved from the protein data bank (PDB) accession 4IEM. The amino acids discussed in this study are labeled and shown in a stick representation (B). (**C**) Cell viability at increasing times after transfection of HeLa cells overexpressing each APE1 variant was determined by colorimetric (MTS) assay. Data represent the mean ± SD of three independent experiments. (**D**) Expression of L104R, R237C and D283G negatively affects APE1 protein-protein interaction. Representative Western blot analysis on co-immunoprecipitated proteins from HeLa cells overexpressing APE1 genetic variants. Co-immunoprecipitated proteins were detected by Western blotting using specific antibodies, as indicated on the right-hand side. FLAG was used as loading control (top). Histograms report the normalized values for the association of the different APE1 variants (horizontal axis) with the protein interacting partners. APE1^WT^ was used as a reference (by attributing a 100% interaction value). Mean ± SD values are the results of three independent experimental sets (bottom). Student's *t*-test calculation was performed on three independent experiments to assess the significance between APE1^WT^ and genetic variants, as indicated; *p ≤ 0.05; ns, non-significant. IP, immunoprecipitate. (**E**) AP endonuclease incision activity of APE1 genetic variants. Relative AP-incision efficiency in time-dependent kinetics for APE1^WT^ and variant APE1 immunoprecipitates. Mean ± SD values are the results of three independent experimental sets (top). Student's *t*-test calculation was performed on three independent experiments to assess the significance between APE1^WT^ and genetic variants, as indicated; *p ≤ 0.05; ns, non-significant. A Western blot analysis on APE1 immunoprecipitates was used for normalization of the results from AP endonuclease incision assays (bottom).

To guide functional characterisation, we evaluated the possible impact of a subgroup of APE1 polymorphisms (L104R, D148E, R237C and D283G) with respect to properties expected to affect protein structure and/or function; analysis was realized with four computational methods (PROVEAN [16], SIFT [17], PolyPhen-2 [18] and CUPSAT [19]). All the modeling algorithms (Figure 1A–1B and Table 1) predicted that 3 amino acid substitutions (i.e. L104R, R237C and D283G) would have an overall protein destabilizing effect or otherwise should affect APE1 function (Table 1). The other polymorphism (i.e. D148E), although destabilizing, was considered to be tolerated and benign (Table 1). In agreement with these predictions, Hadi and colleagues and Illuzzi *et al.* previously demonstrated that D148E and L104R mutations do not show any altered AP-endonuclease activity *in vitro* [8, 9]. Interestingly, D148E and L104R have been shown to display strongly impaired 3′-RNA phosphatase and endoribonuclease activities, while their endonuclease activity towards DNA appeared unaffected [15, 20]. The R237C substitution was previously associated with a diminished APE1 activity *in vitro* [8] and it has been recently found to present reduced incision capacity in proximity of nucleosomes and at pre-assembled DNA glycosylase/AP-DNA complexes [21].

### Unaltered subcellular distribution of APE1 genetic variants

None of the residues associated with these polymorphic variants is a candidate site for PTMs. Initial experiments were aimed at characterizing the above-mentioned APE1 mutants in terms of protein expression level and subcellular distribution. To test a potential impact on intracellular localization, HeLa cells transiently transfected with plasmids expressing Flag-tagged APE1 variants were analyzed by immunofluorescence microscopy. All the variants displayed a subcellular distribution pattern comparable to that of the wild-type protein, with a predominant nucleoplasmic localization and an apparent nucleolar accumulation (Supplementary Figure S1), as previously shown by Illuzzi *et al.* [8].

To assess whether the expression of these APE1 variants may impact on cell viability, we measured the fraction of living cells at 48, 72 and 96 h after transfection through the MTS assay. Expression of D148E and R237C variants resulted in a cell viability comparable to that of cells expressing wild-type APE1 (APE1^WT^), whereas cells expressing the L104R variant had a somewhat increased viability at 96 h (Figure 1C). On this basis, these variants do not seem to negatively impact on cell survival when co-expressed with the endogenous APE1 protein. Hence, the variants do not appear to act as dominant negative alleles. With the exception of the D148E mutant, however, all variants had shorter half-lives compared to the ectopically expressed wild-type counterpart, when co-expressed with the endogenous APE1 (Supplementary Figure S2). This observation suggests that the presence of the endogenous protein may possibly mask an eventual negative impact of these variants.

### APE1 genetic variants have reduced ability to form BER-competent complexes in HeLa cells

The different steps of the BER pathway are highly coordinated. XRCC1 protein serves as a scaffold to hand over the substrates between APE1 and the next enzyme in the BER pathway, *i.e.* Polβ. Thus, we tested whether the ability of APE1 to engage a proper protein-protein interaction with XRCC1 and Polβ was impaired as result of protein polymorphisms. Four variants were selected for further analyses, based on the prediction that three of these mutants (L104R, R237C and D283G) were expected to impact on APE1 function, whereas D148E was predicted being tolerated and benign (Table 1).

Co-immunoprecipitation analyses were performed in HeLa cells transiently transfected with Flag-tagged APE1 expressing plasmids; the amounts of Polβ and XRCC1 immunoprecipitated together with each APE1 variant was determined after normalization with respect to the corresponding FLAG-tagged variant levels before immunoprecipitation. The variant predicted to be tolerated, D148E, interacted with Polβ and XRCC1 as efficiently as APE1^WT^. The variants (L104R, R237C and D283G) predicted to impact on APE1 function showed a reduced ability to pull down Polβ and XRCC1 from the extracts (Figure 1D and Supplementary Figure S3), strongly suggesting a corresponding reduced capability to form BER complexes in cell culture.

To test whether the AP-endonuclease activity of these immunopurified APE1 complexes was reduced, we used a DY782-labeled duplex DNA substrate that harbors a tetrahydrofuran residue mimicking a single abasic site at the central position [22]. In kinetics experiments (Figure 1E and Supplementary Figure S4A), the BER complex pulled down with APE1^WT^ converted about 60% of the substrate within 10 min. In contrast, the complexes formed with R237C and L104R variants were able to cleave about 20% and 40% of the substrate, respectively. In agreement with a previous report [8], the D148E mutant presented no reduction in cleavage activity (Figure 1E and Supplementary Figure S4A). The D283G variant had no detectable cleavage activity under these assay conditions. However, measurements of product formation at increasing amounts of the D283G-immunoprecipitated BER-complex (Supplementary Figure S4B) confirmed that this variant is not a loss of function mutant; it simply showed a dramatically reduced activity, consistent with the requirement of the negatively-charged aspartate residue for efficient catalysis [9, 23].

### Expression of APE1 genetic variants in HeLa cells impairs cell viability and growth

Previous evaluation of the possible functional implications of APE1 variants, by using recombinant purified proteins in *in vitro* assays, showed no consistent correlation between APE1 activity and viability of cells transfected with the corresponding plasmids [8, 9]. One caveat of these experiments was that the possibility of a complementation effect provided by the presence of the endogenous APE1 protein could not be *a priori* excluded. The reduced half-life of these variants (Supplementary Figure S2) further suggested a possible masking effect of any deleterious phenotype associated with their expression.

**Figure 2:**
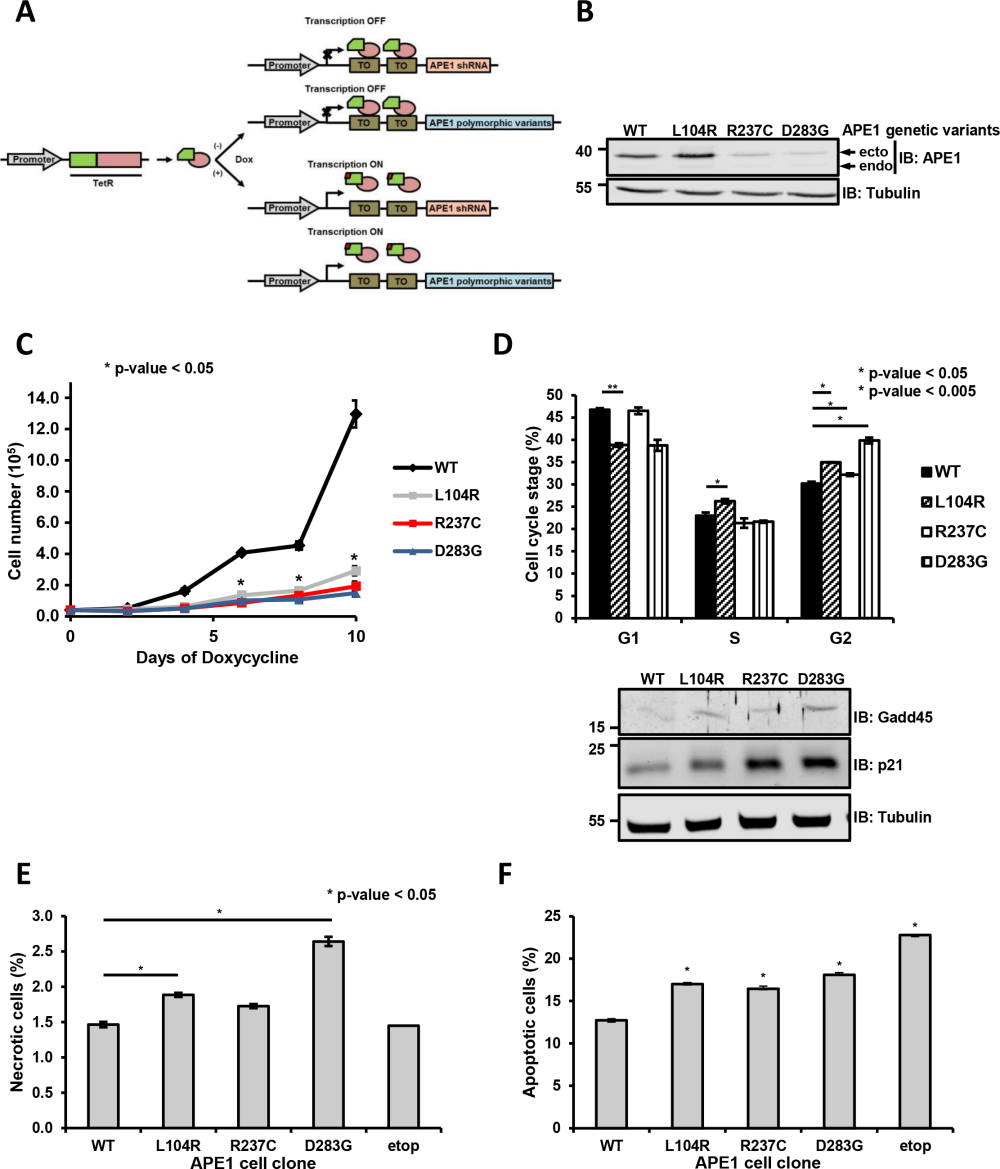
Expression of APE1 variants results in a reduced cell viability and growth defects (**A**) Knock-in strategy for the generation of APE1 genetic variants stable cell clones. HeLa cells were used as a general cellular model and subjected to reiterative transfection cycles for the stable acquisition in series of: a) a Tet repressor constitutively expressed; b) a specific APE1 shRNA under the control of a doxycycline-responsive promoter; c) shRNA-resistant FLAG-tagged APE1 genetic variants under the control of a doxycycline-responsive promoter. Adding doxycycline to the culture medium allowed the expression of the APE1 shRNA, with the subsequent silencing of the endogenous protein and the concomitant expression of the ectopic counterpart. (**B**) Suppression of endogenous APE1 and expression of APE1 genetic variants in HeLa stable cell clones. Representative Western blot analysis of APE1 genetic variants from stable cell clones silenced for endogenous APE1. Two clones for each polymorphism were assayed (data not shown). Levels of endogenous (endo) or ectopic (ecto) proteins were detected with specific antibodies, as indicated on the right-hand side. Tubulin was used as loading control. (**C**) Clones expressing APE1 genetic variants have a growth defect. Growth was followed through Trypan blue staining by measuring cell numbers at the indicated times upon doxycycline treatment. Data, expressed as cell number, are the mean ± SD of three independent experiments. Student's *t*-test calculation on three independent experiments was performed to assess the significance between APE1^WT^ and genetic variants, as indicated; *p ≤ 0.05. (**D**) Cell cycle distribution. Analysis of the distribution into various stages of the cell cycle in clones expressing APE1 genetic variants after 9 days of doxycycline treatment. G1, S and G2 phases are indicated. Mean ± SD values are the results of three independent experiments. Below, Western blots showing increased expression of cell cycle arresting regulators p21 and Gadd45 in whole cell extracts of APE1 cell clones. Tubulin was used as loading control. (**E** and **F**) Cell necrosis and apoptosis. To measure the fraction of apoptotic and necrotic cells, clones expressing APE1 genetic variants were grown for 9 days with doxycycline, labeled with Annexin V FITC and PI to monitor apoptosis and necrosis, respectively, using FACS analysis. The fraction (%) of necrotic (E) and apoptotic (F) cells is plotted in histograms. Bar graph shows the average of 3 independent experiments ± SD. Asterisks represent a significant difference (*p* ≤ 0.05) with respect to APE1^WT^. As positive control for apoptosis, we used clones expressing APE1^WT^ treated with 200 μM etoposide for 24 h (etop).

To exclude this experimental bias, we established an experimental reconstitution strategy where the endogenous protein was silenced using small-hairpin (sh) RNA technology, concomitantly with the expression of shRNA-resistant APE1 variants (Figure 2A and 2B) [24]. Upon treatment with doxycycline for 9 days, we achieved an efficient silencing of the endogenous APE1 protein, with less than 10% residual expression (Figure 2B). All the subsequent analyses were performed in at least two independent clones for each APE1 variant. In agreement with the transient overexpression experiments and the measured reduced half-life, the expression levels of the different variants varied, with two mutants (R237C and D283G) showing lower expression levels than the ectopically expressed APE1^WT^ (Supplementary Figure S2, *top*). Whereas the expression of APE1^WT^ was well tolerated in this system, the growth of clones expressing the variants was impaired, as evident from measuring the corresponding growth rate by cell counting (Figure 2C) or colony-forming ability (Supplementary Figure S5A). No correlation was observed between growth impairment and expression level of the different variants; in fact, no significant differences in the growth rate of clones expressing L104R, R237C and D283G variants were measured. Since low levels of APE1 expression were previously demonstrated to sustain cell growth [25], the reduced growing we observed seemed likely associated with the expressed variant *per se*. Moreover, the measured slow growth rates were associated with a slight increased G2- and S-phase permanence, especially for L104R- and D283G-expressing cells (Figure 2D, *top*). Within the cycling population, a decrease in the ratio between cells in the G1 and S+G2 phases was detected (G1/S+G2 ratio: WT, 0.88; L104R, 0.64; R237C, 0.87; D283G, 0.63), suggesting a possible minor impairment in the passage to the following G1-phase, due to the expression of these APE1 variants. In accordance, Western blot analyses showed p21 and Gadd45 stabilization under basal conditions in the clones expressing APE1 genetic variants, thus suggesting an induced check point activation under basal conditions (Figure 2D, *bottom*). The altered proliferation indexes observed for all the genetic variants were accompanied by increased fractions of necrotic (Figure 2E) and apoptotic (Figure 2F) cells under basal conditions, as measured by FACS analysis (Supplementary Figure S5B).

### Expression of APE1 genetic variants leads to impaired rRNA biogenesis and induction of autophagy

**Figure 3:**
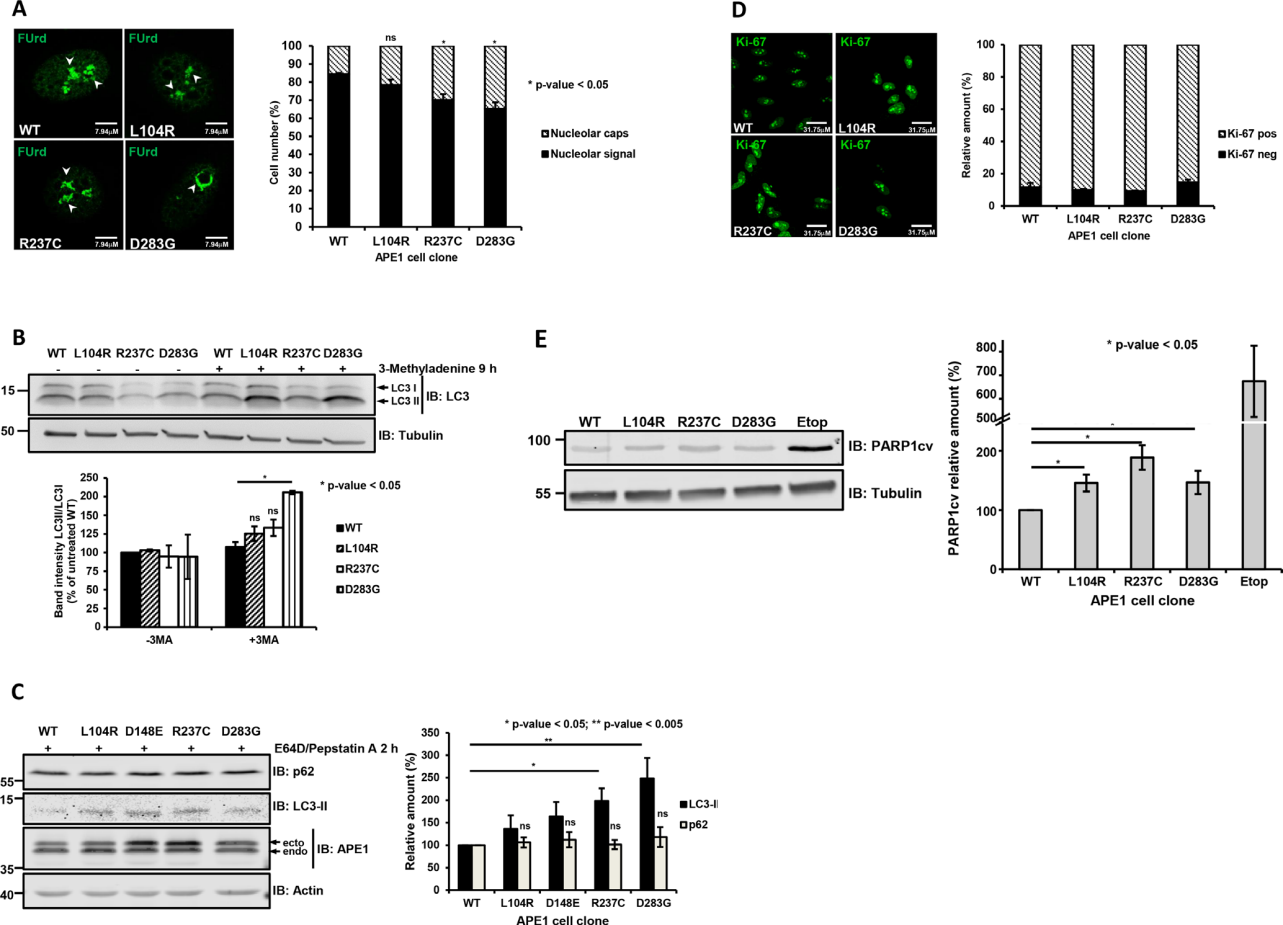
Impaired rRNA biogenesis and induction of autophagy in HeLa cells expressing APE1 genetic variants (**A**) Expression of R237C and D283G variants leads to nucleolar stress. FUrd labeling of reconstituted cell lines 9 days after doxycycline treatment. Representative immunofluorescence images showing impaired FUrd accumulation and preferential nucleolarcap formation (left). Histogram reporting percentage of cells with FUrd nucleolar incorporation and nucleolar caps in the different clones (right). Bar graph shows the average of 3 independent experiments ± SD. *p ≤ 0.05; ns, non-significant. (**B**) Induction of autophagy. Western blots showing autophagy induction in APE1 cell clones by monitoring LC3-I to LC3-II conversion in the absence (−) or presence (+) of 3-methyladenine (5 mM for 9 h) in whole cell extracts. Tubulin was used as loading control (top). LC3-I and LC3-II bands were normalized to those of tubulin; relative LC3-II/LC3-I levels in the different clones were determined after normalization to corresponding value of APE1^WT^ (bottom). Bar graph shows the average of 3 independent experiments ± SD. *p ≤ 0.05; ns, non-significant. (**C**) R237C and D283G variants induce autophagy in transient transfected HeLa cells. Western blots showing autophagy induction in transiently transfected HeLa cells expressing APE1 genetic variants through the monitoring of LC3-II and p62 levels in whole cell extracts. Cells were treated with E64D (10 μg/ml) and pepstatin A (10 μg/ml) for 2 h before harvesting. Levels of endogenous (endo) or ectopic (ecto) proteins were detected with specific antibodies, as indicated on the right-hand side. Actin was used as loading control (left). LC3-II and p62 bands were normalized to those of tubulin; relative quantification of LC3-II and p62 levels in the different clones was determined after normalization to corresponding values of APE1^WT^ (right). Student's *t*-test calculation on three independent experiments was performed to assess the significance between APE1^WT^ and genetic variants, as indicated; *p ≤ 0.05; **p ≤ 0.005; ns, non-significant. (**D**) Cellular senescence does not contribute to growth defect. Assessment of Ki-67 as marker of senescence in APE1 variants-reconstituted cell lines after 8 days of doxycycline treatment. Representative immunofluorescence images of Ki-67 (left). Mean of quantitative estimates of Ki-67 negative cell fraction within the clones (right). Bar graph shows the average of 3 independent experiments ± SD. (**E**) Expression of APE1 variants leads to an increased basal PARP1 cleavage. Representative Western blots showing cleaved PARP1 (PARP1cv) in cell lines reconstituted with APE1 genetic variants. Tubulin was used as loading control (left). PARP1cv levels were quantified and normalized to the corresponding values of APE1^WT^ (right). As positive control for PARP1 cleavage, we used clones expressing APE1^WT^ treated with 200 μM etoposide for 24 h (etop).

As the minor perturbations observed in the cell-cycle progression were unlikely in explaining the observed reduction in growth, we tested whether the expression of these variants was impacting on other cell parameters related to APE1 functions. To test whether expression of APE1 mutants in APE1 expressing cell clones interfered with ribosome biogenesis [12, 26–28], we assessed the corresponding nucleolar incorporation of the fluorinated UTP analogue fluorouridine (FUrd) into nascent rRNA transcripts [26, 29, 30]. Expression of these genetic APE1 variants resulted in a reduced nucleolar FUrd uptake (Figure 3A), which is a phenotype associated with nucleolar stress. Moreover, expression of the R237C and D283G variants led to the formation of nucleolar ring structures (Figure 3A, *right*), which are typical indications of autophagic activity in the nucleolar caps [31–33]. Thus, we monitored the induction of autophagy in clones reconstituted with APE1 variants by following LC3-I to LC-II conversion after treatment with 3-methyladenine (Figure 3B) and LC3 puncta formation (Supplementary Figure S6A). Silencing of APE1 itself resulted in a reduction of p62 levels, but it was not associated with a significant increase of the LC3 cleavage (Supplementary Figure S6B). Reconstitution of these cells by the expression the APE1 variants resulted in a weak, though consistent, increase in LC3-II cleavage (Figure 3B), in agreement with an induction of autophagy. To exclude any bias due to the possible onset of adaptive phenomena in the reconstituted clones, we monitored p62 levels and lysosomal turnover of LC3 in transiently transfected HeLa cells treated with the lysosomal protease inhibitors E64D and pepstatin A [34]. Expression of the APE1 variants resulted in a 1.5 to 2.5-fold increase in LC3-II formation, compared to cells expressing APE1^WT^, while no marked accumulation of p62 was observed (Figure 3C). These data confirmed the observed increased autophagic flux in cells expressing these APE1 variants. To assess the activation of other pathways that may negatively impact on cell growth [35, 36], we monitored the senescence Ki-67 marker upon expression of these APE1 variants [37]; no significant changes were observed in this case (Figure 3D). On the other hand, a mild induction of the apoptotic program, as measured by PARP1 cleavage (PARP1cv) (Figure 3E) and by previous FACS analysis (Figure 2F), was observed in all clones and in cells transiently transfected with these APE1 variants (Supplementary Figure S6C). On this basis, a combined occurrence of slow cell-cycle progression, induction of autophagy and apoptotic/necrotic events was claimed to account for the slow-growing phenotype of cells expressing the above-mentioned APE1 genetic variants.

### APE1 genetic variants showed altered resistance to genotoxic damages

**Figure 4:**
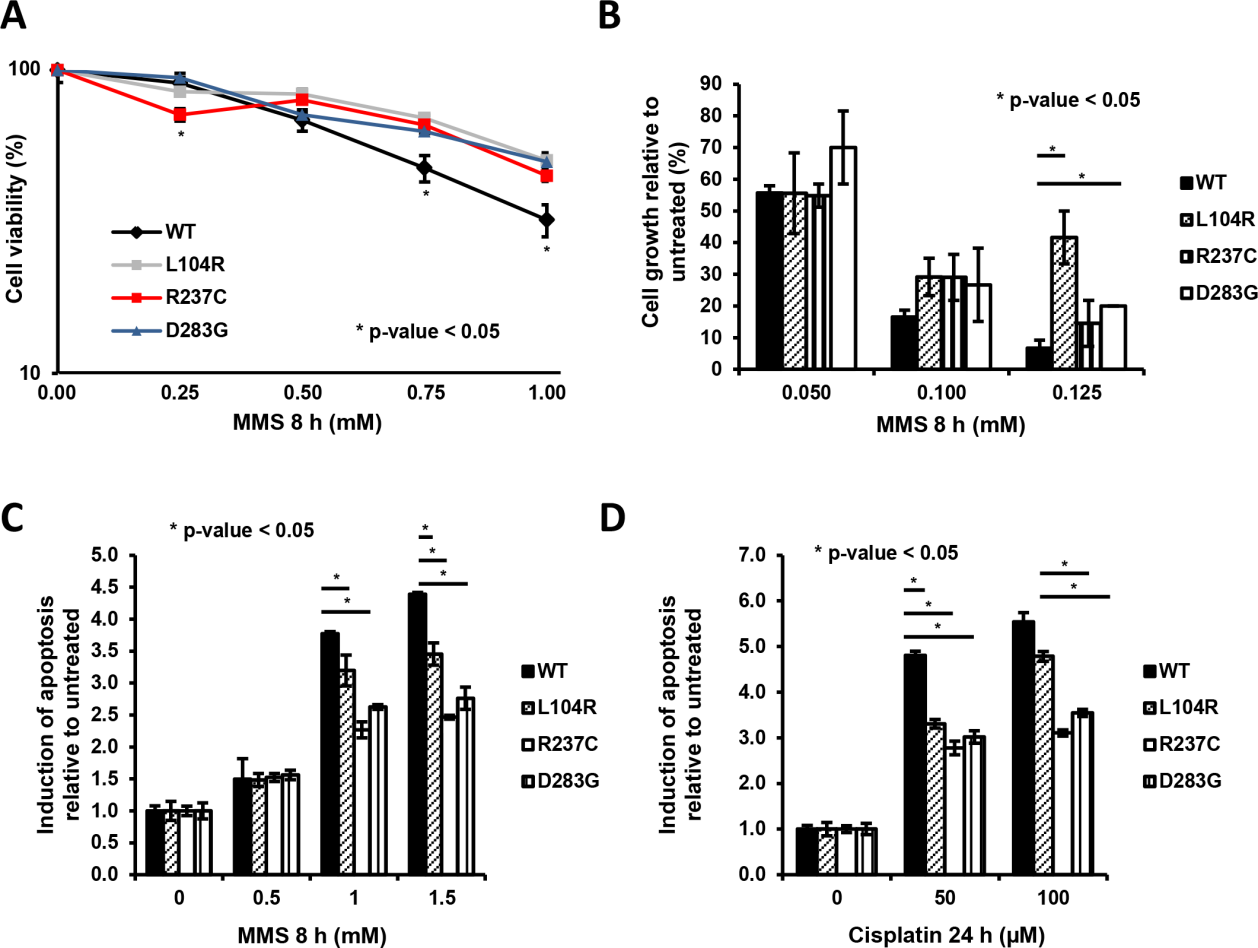
Clones expressing APE1 genetic variants are less sensitive towards genotoxic damage (**A**) Cell viability of APE1 variants-reconstituted clones subjected to treatment with increasing amounts of MMS was determined by a colorimetric (MTS) assay. Data presented using a logarithmic scale showing the mean ± SD of three independent experiments. (**B**) Cell growth of APE1 variants-reconstituted clones subjected to treatment with increasing amounts of MMS was measured by the colony formation assay. Data, expressed as the percentage of change with respect to untreated clones, are the mean ± SD of three independent experiments. (**C** and **D**) Clones expressing APE1 genetic variants were grown for 9 days with doxycycline and treated with increasing doses of MMS for 8 h (C) and cisplatin for 24 h (D), as indicated. Apoptosis was assayed with PI staining and is reported as fold change of induction with respect to each untreated clone. Bar graph shows the average fold of induction of apoptosis in 3 independent experiments ± SD, as normalized with respect to untreated clones. Asterisks represent a significant difference respect to APE1^WT^.

As these APE1 genetic variants had reduced capacity to form BER-competent complexes and showed reduced AP-endonuclease activity (Figure 1D and 1E and Supplementary Figure S4A), we tested whether the cells expressing these mutants were sensitive to MMS, an agent generating lesions that are repaired by BER [38–41]. Contrary to all expectations, MTS assays demonstrated that all the clones expressing APE1 variants were less sensitive to MMS, compared to cells expressing APE1^WT^ (Figure 4A). The protective effect towards MMS was confirmed using the colony formation assay (Figure 4B) and FACS analysis (Figure 4C), showing an increased fraction of apoptotic cells in APE1^WT^-expressing clones. A similar effect was also observed in cells treated with cisplatin, a DNA crosslinking agent generating ROS and oxidative DNA lesions that are repaired through BER, with the essential involvement of APE1 [39, 41–44] (Figure 4D and Supplementary Figure S7A–S7B). Thus, expression of APE1 variants did not result in hypersensitivity to genotoxins. Instead, a mild tolerance to DNA damaging agents was observed that is likely associated with the slow growing phenotype or with the nucleolar stress induced by these agents.

### Increased poly(ADP-ribosyl)ation and DNA damage levels in APE1 genetic variants

**Figure 5:**
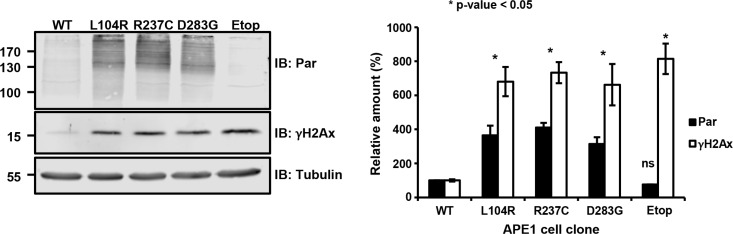
Expression of APE1 variants leads to a basal activation of the DNA damage response Representative Western blots showing PAR protein and γH2Ax levels in APE1 genetic variant-reconstituted cell lines upon doxycycline treatment. Tubulin was used as loading control (left). Quantification of PAR protein and γH2Ax levels was obtained after normalization to APE1^WT^ (right). As positive control, we measured PAR protein and γH2Ax levels in clones expressing APE1^WT^ treated with 200 μM etoposide for 24 h (etop). All the clones displayed a statistically significant change (*p* ≤ 0.05) in PAR protein and γH2Ax levels, considering APE1^WT^ as reference. Bar graph shows the average of 3 independent experiments ± SD.

In the above, we have shown that the expression of APE1 variants leads to growth defects which are accompanied by slow cell-cycle progression, nucleolar stress, induction of autophagy and an increased basal activation of apoptosis. These phenotypes may be linked to and be suggestive for a condition of a cellular stress as a consequence of inefficient processing of endogenous DNA damage. To test this hypothesis, we measured the levels of two well-established markers of DNA damage response activation, γH2Ax and poly(ADP-ribosyl)ated (PAR) proteins, in clones re-expressing these APE1 variants. In this context, PAR proteins are considered as biomarkers of PARP1 activation [45]. All the genetic variants displayed a robust increase in both markers, when compared to clones expressing APE1^WT^ (Figure 5). In particular, PAR levels were increased about 8-fold and much higher than counterparts measured in cells treated with etoposide (Etop); similarly, γH2Ax levels were increased about 4-fold (Figure 5). Analogous results were observed upon transient expression of these variants in HeLa cells (Supplementary Figure S8). Thus, the expression of these APE1 mutants seemed to elicit a chronic induction of DDR signaling, which may explain the slow cell-cycle progression and growth defects we observed. Importantly, these phenotypes were observed in cells in the absence of genotoxin exposure, emphasizing the importance of efficient BER to protect cells against endogenous DNA damage. The phenotypes activated in response to this stress likely contribute to a tolerance to additional stressors (Figure 4). The chronic genomic stress induced by the expression of APE1 variants, resulting in persistent activation of the DDR, would also be expected to set up a selective pressure towards loss of DNA damage checkpoint mechanisms, thus suggesting a rationale for the seemingly paradoxical slow-growing phenotype observed in cell culture and an eventual association with human cancer.

## DISCUSSION

Recent studies have identified correlations between defects in APE1 and predisposition to human diseases, including cancer [5]. Although severe deficiencies in APE1 are incompatible with life in mammals, several APE1 SNPs have been identified [5], but epidemiological studies have reported conflicting associations with disease. *In vitro* data are available for functional and structural consequences of several APE1 variants [8, 15]. In this study, we provide evidence for a biological effect in cultured cells of some of these mutations.

We characterized the molecular and cellular phenotypes arising as a result of the expression of a group of APE1 genetic variants (summarized in Table 2). Except for the D283G mutant, these variants involve amino acids that do not appear directly responsible for either APE1 AP-endonuclease activity or its redox-regulatory function. These mutants presented a nuclear subcellular localization indistinguishable from that of APE1^WT^, demonstrating that none of these amino acid substitutions affects the distribution of the protein itself. However, we observed a marked reduced cell growth in clones expressing these APE1 variants, where the expression of the endogenous protein was suppressed using shRNA. These conditions recapitulate a heterozygous situation, where APE1 variants are observed in human population. In experimental models we set up for mimicking a heterozygous or homozygous situation, the expression of these variants caused the formation of nucleolar caps structures and other markers associated with autophagic activation. On this basis, it is possible to hypothesize that, concomitantly with mutant expression, proteins associated with damaged DNA are removed from the nucleolus via autophagy, as recently suggested for other DNA repair proteins [46, 47]. However, further studies would have to confirm whether this is the case for these APE1 variants or whether the induction of autophagy is a consequence of an increased endogenous stress.

**Table 2:** Effect of APE1 genetic variants described in this study on various molecular and cellular parameters

APE1geneticvariant	Predicted effects			Interactome network	Biological outcome				
	Loss of AP-endo activity	Loss of structure stability	Destabilizing overalleffects	BERproteins	Loss of AP-endo activity	Cellcycleperturbation	Cellviabilityduringstressconditions	Autophagyinduction	DDRactivation
**L104R**	**+**	**+**	**+**	**↓**	**+**	**+**	**+**	**+**	**+**
**D148E**	**−**	**−**	**−**	**−**	**−**	N.D.	N.D.	**+**	**+**
**R237C**	**++**	**+**	**+**	**↓**	**++**	**−**	**+**	**++**	**++**
**D283G**	**+++**	**+**	**+**	**↓**	**+++**	**+**	**+**	**+++**	**+++**

N.D., not determined.

Evidence of elevated levels of endogenous genotoxic stress was observed in both heterozygous and homozygous model. Augmented γH2AX levels and increased PARP1 activation were consistent with the induction of replicative stresses due to an inefficient repair of endogenous DNA damage through the BER pathway. Such an effect would explain the slow-growing phenotype we observed [48, 49] and the seemingly contradictory finding of tolerance to treatment with genotoxic agents.

An impaired APE1 endonuclease activity and a consequent reduced BER capacity were observed also in variants not associated with cancer [43]. APE1 also contributes to the regulation of cellular responses to oxidative stress and has other non-DNA repair activities, such as the regulation of the expression of genes involved in chemo-resistance and tumor progression through transcriptional and post-transcriptional mechanisms [50]. Through its redox activity towards different cancer-related transcription factors, APE1 is also involved in the regulation of inflammatory and metastatic processes [50]. Thus, factors not directly related to BER function are likely to shape the phenotypes associated with APE1 variants. Control of APE1 functions is multi-layered and involves PTMs and complex formation with APE1-associated proteins. Hence, a reasonable explanation for the reduced AP-endonuclease activity and for the phenotypes observed after the expression of these APE1 variants may be ascribed to an alteration of the corresponding protein interactomes. An impact for these polymorphisms on APE1 ribosomal function(s) cannot be excluded. By shaping the corresponding protein interactome beyond the BER-interactome studied here, APE1 amino acid substitutions can significantly affect the corresponding biological effects. An active role for APE1 and, possibly, for other BER enzymes, in the regulation of ribosome biogenesis [26, 51] would likely also affect cell sensitivity to DNA damaging agents.

However, the phenotypes described here are all consistent with an interpretation that above-mentioned APE1 variants have a reduced ability to engage BER competent complexes. PARP1 activation and increased basal phosphorylation of H2AX indicated that this impaired BER function was eliciting a situation of chronic endogenous genotoxic stress. We here propose that the persistent activation of PARP1 and the accumulation of DNA damage beyond a certain threshold of physiological DNA repair limit, as well as the induction of autophagy, might create a positive niche for tolerance phenomena and the establishment of cancer cells. This background activation of the DDR may also create an evolutionary pressure that might affect cancer susceptibility in cells expressing APE1 variants.

## MATERIALS AND METHODS

### Inducible APE1 knock-down and generation of APE1 knock-in cell lines

Inducible silencing of endogenous APE1 and reconstitution with APE1 genetic variants in HeLa cell clones was performed as already described [14] and detailed in Supplementary Information. Cell clones were grown for 9 days as indicated [14]. All biological data were reproduced at least in two different cell clones for each APE1 variant.

### Cell culture, treatments and plasmid transfection

HeLa cells were grown in Dulbecco's modified Eagle's medium (DMEM) (Invitrogen, Carlsbad, CA) supplemented with 10% foetal bovine serum (Euroclone, Milan, Italy), 100 U/ml penicillin, and 10 μg/ml streptomycin sulfate. Cisplatin was freshly solved in dimethylformamide before use. Pepstatin A and E64D were purchased from Vinci-Biochem (Florence, Italy) and solved in dimethyl sulfoxide. 3-Methyladenine and bafilomycin A were purchased from Sigma-Aldrich (Oslo, Norway) and solved in water and dimethyl sulfoxide, respectively. Cell transfection was performed as described previously [52]. All chemical reagents were supplied from Sigma-Aldrich (Milan, Italy) unless otherwise specified.

### Preparation of the cell extracts and Western blotting

Whole cell extracts for Western blotting analyses were prepared as previously described [12]. Membranes were developed by using the ECL enhanced chemiluminescence procedure (GE Healthcare, Piscataway, NJ) or by using the NIR Fluorescence technology (LI-COR GmbH, Germany), as indicated in each figure capture. Images were acquired and quantified by using a Chemidoc XRS video densitometer (Bio-Rad, Hercules, CA) or an Odyssey CLx Infrared Imaging system (LI-COR GmbH, Germany). A list of antibodies used is given in the Supplementary Information.

### Co-immunoprecipitation

Co-immunoprecipitation analyses were performed as already described [14, 26].

### APE1 plasmid constructs

Expression constructs for human APE1 variants were created using the QuikChange II Site-Directed Mutagenesis Kit (Stratagene, Santa Clara, CA). Forward and reverse primers containing the relevant nucleotide change were generated following the mutagenic primer design guidelines of the manufacturer. All the mutants were confirmed by DNA sequencing (MWG, Ebersberg, Germany).

### Cell viability, cell growth and clonogeneic assays

Cell viability was measured through the MTS assay (CellTiter 96 AQueous One Solution Cell Proliferation Assay; Promega, Madison, WI) according to manufacturer's instructions. Cell growth and clonogenic assays were performed as described previously [12, 53].

### Assessment of nucleolar transcription

Fluorouridine (FUrd) incorporation was assessed as described previously [26, 29, 30].

### Cell-cycle and apoptotic analysis

Following doxycycline treatment, HeLa cells reconstituted with the APE1 variants were harvested, washed once with ice-cold PBS and fixed at 1 × 10^6^ cells/ml in cold 70% ethanol, at 4°C, overnight. Cells were centrifuged at 200 × *g* for 10 min, at 4°C, and washed twice with ice-cold PBS. Cell pellets were resuspended in a sodium azide/PBS solution containing propidium iodide (PI) (0.04 mg/mL) (Invitrogen, Carlsbad, CA, USA), DNase free RNase A (0.2 mg/mL) (Sigma-Aldrich, Milan, Italy) and 0.1% w/v Triton X-100, and then incubated for 15 min, at 37°C, in the dark, with gentle mixing every 5 min. For apoptotic analysis in response to MMS and cisplatin treatment, cells were trypsinized, washed once with ice-cold PBS and immediately resuspended in PBS/sodium azide solution containing PI (0.04 mg/mL propidium iodide) (Invitrogen, Carlsbad, CA, USA). For the evaluation of necrosis and apotosis, cells were doubly stained using Annexin V-FITC and PI (Apoptosis Detection Kit; eBioscience, # BMS500FI) according to manufacturer's instructions. Cell cycle analyses and quantification of apoptotic cells were performed by flow cytometric analysis using a FACScan (Becton Dickinson, Franklin Lakes, NJ, USA). For each sample, 25,000 single events/cells were detected and data analysis was performed using FCS Express 4 Plus research edition software (De Novo Software, Los Angeles, CA).

### AP-incision assay

AP-nicking assays were performed essentially as previously described [14]. To measure AP-endonuclease activity, 0.75 pmol of 5′DY782 26F DNA oligonucleotide were reacted with 0.75 fmol of APE1^WT^ or variant APE1 immunoprecipitates in 50 mM HEPES, 50 mM KCl, 10 mM MgCl_2_, 1 μg/ml bovine serum albumin and 0.05% w/v Triton X-100 at 37°C, for the indicated times. For the dose-response experiments, the indicated amounts of D283G immunoprecipitates were incubated for 10 min, at 37°C. Gels were visualized with an Odyssey CLx Infrared Imaging system (LI-COR GmbH, Germany). The signals of the non-incised substrate (S) and the incision product (P) bands were quantified using Image Studio software (LI-COR GmbH, Germany).

### Statistical analyses

Statistical analyses were performed by using the Student's *t* test. *P* < 0.05 was considered as statistically significant.

## SUPPLEMENTARY MATERIALS FIGURES



## Abbreviations

APE1/Ref-1: Apurinic apyrimidinic endonuclease/Redox effector factor 1
BER: Base Excision Repair
DDR: DNA Damage Response
MMS: Methyl methanesulfonate
PAR: poly(ADP-ribose)
PI: propidium iodide
Polβ: DNA Polymerase β
PTMs: Post-translational modifications
SNPs: Single Nucleotide Polymorphisms
XRCC1: X-ray repair cross-complementing protein 1
