# Supplementary material for: APE1 polymorphic variants cause persistent genomic stress and affect cancer cell proliferation
Source: Oncotarget. 2016 Mar 30;7(18):26293–306. doi: 10.18632/oncotarget.8477 (PMC5041981; doi:10.18632/oncotarget.8477)
Supplement: Supplementary file 1 [file oncotarget-07-26293-s001.pdf]

APE1 polymorphic variants cause persistent genomic stress and affect cancer cell proliferation

Supplementary Materials

SUPPLEMENTARY RESULTS

APE1 variants localize in the nucleoplasm with strong accumulation in the nucleoli, similarly to the wild-type protein

In order to test the potential impact of APE1 L104R, D148E, R237C and D283G mutants on protein intracellular localization, the corresponding transiently-transfected HeLa cells were assayed by immunofluorescence analysis. All the variants displayed a subcellular distribution comparable to that of APE1^{WT}, with a predominant nucleoplasmic localization associated with an apparent nucleolar accumulation (Supplementary Figure S1).

SUPPLEMENTARY INFORMATION ON EXPERIMENTAL PROCEDURES

Inducible APE1 knock-down and generation of APE1 knock-in cell lines

All these procedures have been described previously [1, 2]. For the generation of HeLa reconstituted APE1 variants, APE1 variants expression vector was generated by cloning an *EcoRI-BamHI* fragment from pFLAG-CMV-5.1/APE1 (Sigma-Aldrich, Milan, Italy) into p3XFLAG-CMV-14 vector (Sigma-Aldrich, Milan, Italy). To avoid the degradation of the ectopic APE1 mRNA, two nucleotides of the APE1-cDNA coding sequence were mutated by using the Site-Directed Mutagenesis Kit (Stratagene, Santa Clara, CA), leaving unaffected the APE1 amino acid sequence: siRNA: 5′-CCTGC<u>C</u>AC<u>A</u>CTCAAGATCTGC-3′; APE1: 5′-CCTGC<u>A</u>AC<u>G</u>CTCAAGATCTGC-3′. The Site-Directed Mutagenesis Kit was used to generate the APE1 genetic variants. All the mutants were confirmed by DNA sequencing (MWG, Ebersberg, Germany). Then, the APE1 siRNA clone was transfected with p3XFLAG-CMV/APE1^{WT} and genetic variants, previously digested with *ScaI* (Thermo Scientific, Milan, Italy). Forty-eight h after transfection, the cells were subjected to selection with geneticin (Invitrogen, Carlsbad, CA) for 14 days, and selected for the acquired resistance. Individual cell clones were isolated by using cell cloning cylinders (Sigma-Aldrich, Milan, Italy), transferred and grown for expansion to 10^7 cells in the presence of selective antibiotics. As control, the siRNA control clone was transfected with the p3XFLAG-CMV-14 empty vector. After 10 days of doxycycline treatment at the final concentration of 1 μg/ml, total extracts were analyzed for APE1 expression by immunoblotting, thus revealing the silencing of the endogenous APE1 and the expression of the ectopic flagged wild-type and variants of the protein.

Immunofluorescence and confocal microscopy

For immunofluorescence analyses, cells were plated onto glass coverslips, fixed with 4% w/v paraformaldehyde for 20 min at room temperature and permeabilized with PBS containing 0.1% v/v Triton X-100. Cells were saturated with 10% v/v fetal bovine serum in TBS/0.1% v/v Tween-20 for 1 h, at room temperature, incubated with the α-FLAG antibody (1:200) for 2 h at 37°C, and then washed three times with TBS containing 0.1% Triton X-100, each for 5 min. Alexa Fluor 488–conjugated secondary antibody (Jackson ImmunoResearch, West Grove, PA) was used for detection. DNA was labelled with TO-PRO3® (Sigma-Aldrich, Milan, Italy). Confocal fluorescent images were obtained using a Leica TCS SP laser-scanning confocal microscope (Leica Microsystems, Wetzlar, Germany) equipped with a 488 nm-argon laser, a 543 nm-HeNe laser, and a63 × oil immersion objective (HCX PL APO CS 63 ×/1.32–0.60; Leica). Data were acquired at room temperature (23°C) using the integrated Leica Confocal Software package; multicolor images were captured through sequential scanning.

Antibodies for Western blotting and immunofluorescence

Monoclonal α-APE1 was from Novus (NB 100-116); polyclonal Gadd45 was from Santa Cruz (sc797); monoclonal p21 was from Cell Signaling (#2947); monoclonal α-PARP1cv was from Novus (NB100-56599); monoclonal α-Poly(ADP-ribose) (PAR) was from Adipogen (10H); monoclonal γH2Ax was from Millipore (05-636); monoclonal α-XRCC1 was from Thermo Scientific (MS-434-P0); polyclonal α-Polβ was from Abcam (ab26343); monoclonal α-FLAG was from Sigma (F1804); polyclonal α-actin and monoclonal α-tubulin were from Sigma (A2066 and T-9026, respectively); monoclonal α-p62 was from BD Biosciences (610832) and polyclonal α-LC3 was from Cell Signaling (2775). Polyclonal Ki67 was a kind gift from Dr. Antonio Beltrami.

REFERENCES

1. Lirussi L, Antoniali G, Vascotto C, D'Ambrosio C, Poletto M, Romanello M, Marasco D, Leone M, Quadrifoglio F, Bhakat KK, Scaloni A, Tell G. Nucleolar accumulation of APE1 depends on charged lysine residues that undergo acetylation upon genotoxic stress and modulate its BER activity in cells. Molecular biology of the cell. 2012; 23:4079–4096.

2. Vascotto C, Cesaratto L, Zeef LA, Deganuto M, D'Ambrosio C, Scaloni A, Romanello M, Damante G, Taglialatela G, Delneri D, Kelley MR, Mitra S, Quadrifoglio F, et al. Genome-wide analysis and proteomic studies reveal APE1/Ref-1 multifunctional role in mammalian cells. Proteomics. 2009; 9:1058–1074.

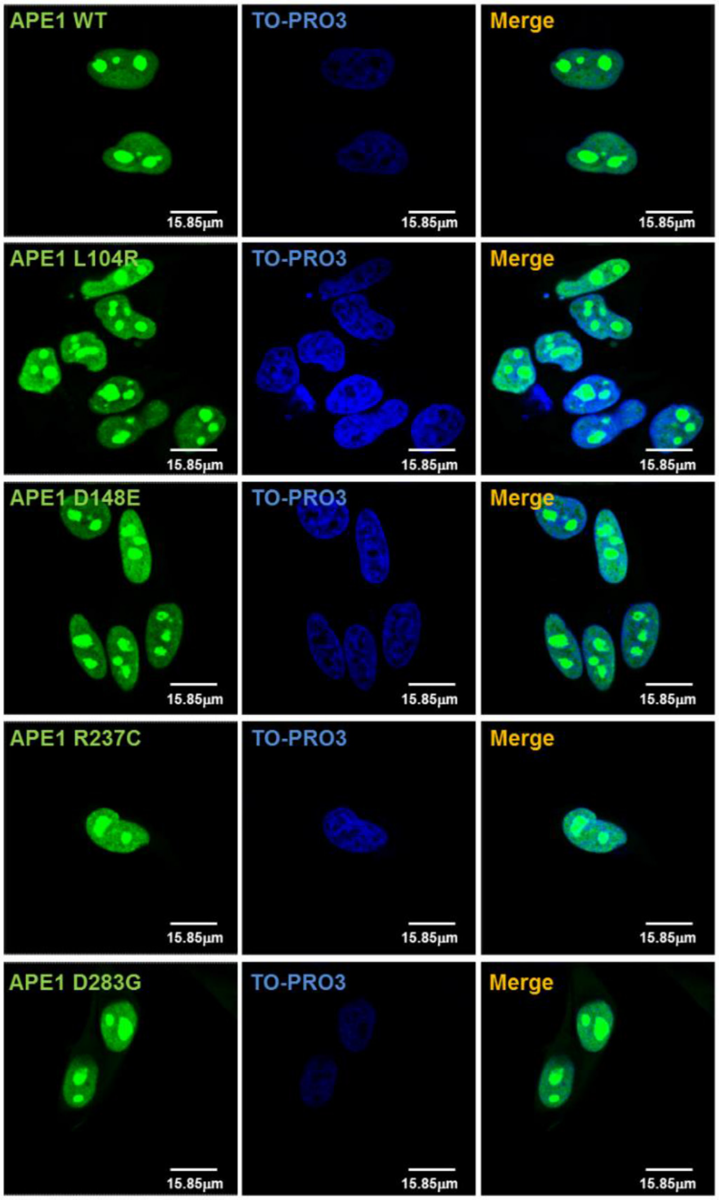

Supplementary Figure S1: APE1 variants localize to the nucleoplasm with strong accumulation in the nucleoli. Representative immunofluorescence panels showing the subcellular localization pattern of the different APE1 variants. Ectopic APE1 staining (FLAG, green) was used to localize HeLa cells positively transfected with the FLAG-tagged APE1 genetic variants constructs. TO-PRO3 counter-staining was used to mark nuclei; size bars correspond to 15.85 μm.

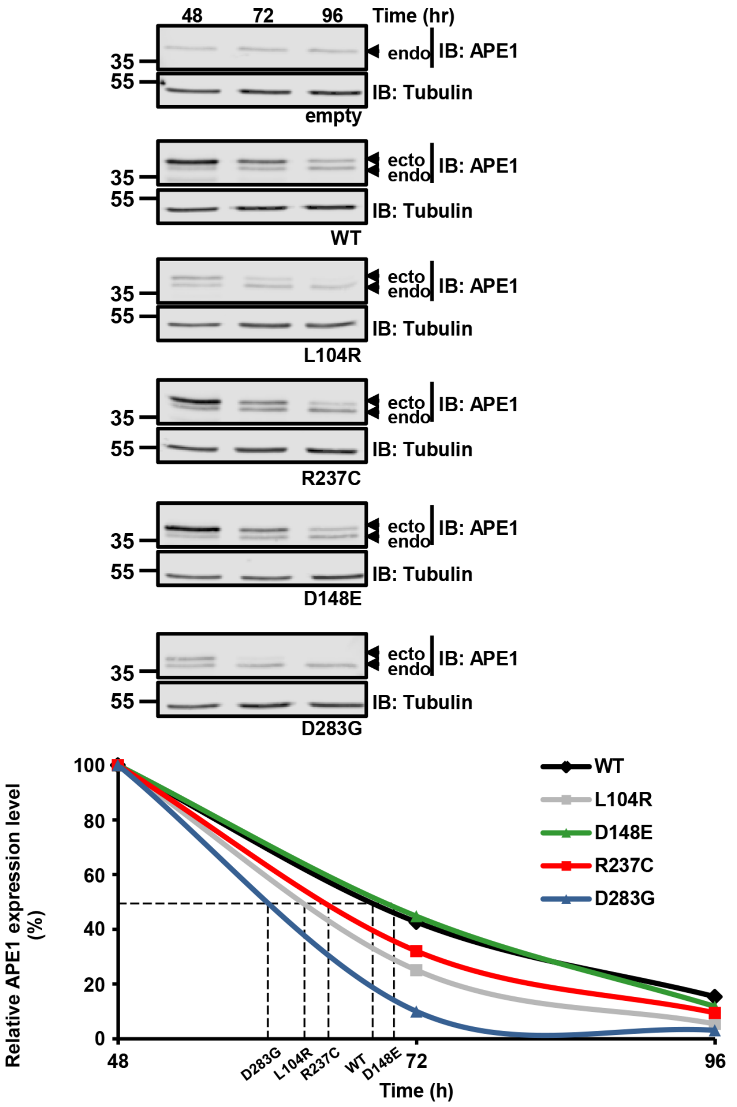

Supplementary Figure S2: Diminished half-life of APE1 variants. Representative Western blotting analysis on HeLa cells overexpressing APE1 variants. Levels of endogenous (endo) or ectopic (ecto) proteins were detected with a specific antibody, as indicated on the right-hand side. Tubulin was used as loading control (top). Protein stability of ectopic APE1 variants was determined at 48, 72 and 96 h after transfection (bottom). Quantification of ectopic APE1 half-life was deduced from the western blots, normalized to tubulin. The fifty percent of ectopic protein is indicated. The experiment was repeated twice. Developed by using the NIR Fluorescence technology.

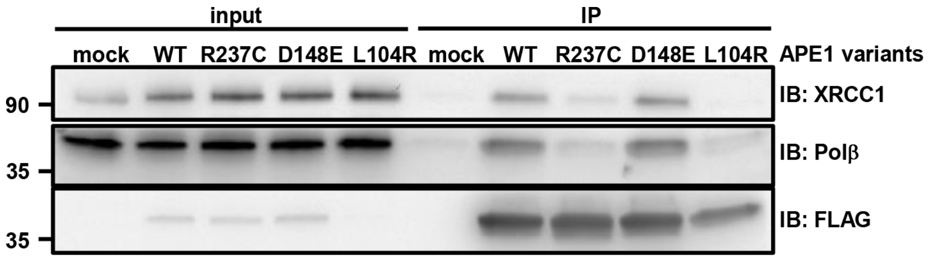

Supplementary Figure S3: APE1 protein-protein interactions are influenced by the expression of APE1 variants. (A) Expression of R237C, D283G and L104R negatively affects APE1 protein-protein interactions. Representative Western blotting analysis on co-immunoprecipitated proteins from HeLa cells overexpressing APE1 variants. Co-immunoprecipitated proteins were detected by Western blotting using specific antibodies, as indicated on the right-hand side. FLAG was used as loading control (top). IP, immunoprecipitate. Membranes were developed using the ECL enhanced chemiluminescence procedure.

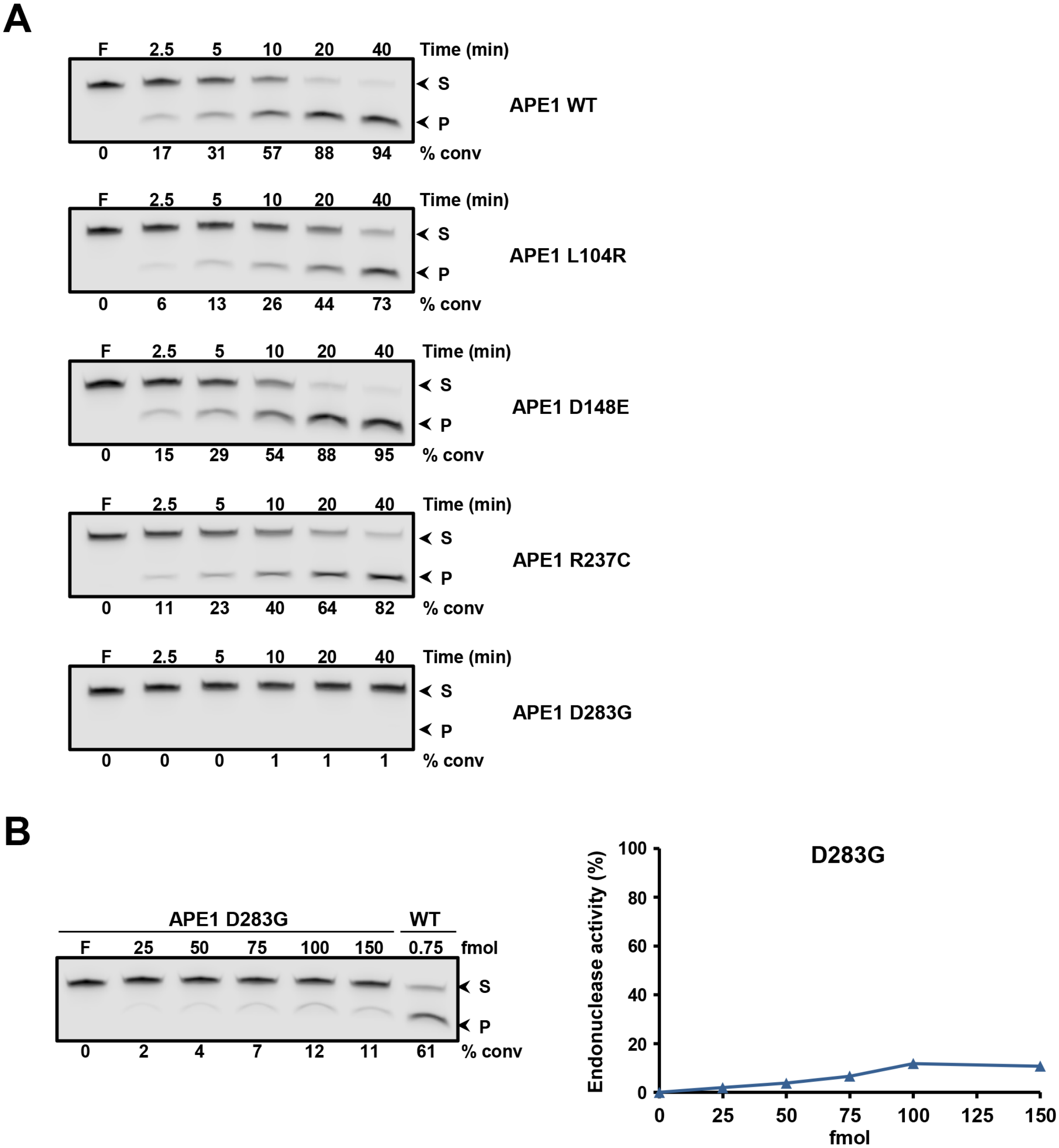

Supplementary Figure S4: AP endonuclease incision activity. (**A**) Representative images of time-dependent kinetics with wild-type and variant APE1 immunoprecipitates, as shown in Figure 1E. Immunoprecipitates (0.75 fmol) were incubated with 5′DY782-labeled 26F DNA substrate and resolved on a urea-polyacrylamide denaturing gel. The non-incised substrate (S) and incision product (P) bands were visualized and quantified using Licor Odyssey. F, no immunoprecipitates. Developed by using the NIR Fluorescence technology. (**B**) Increasing concentrations of D283G and WT immunoprecipitates were incubated for 10 min with 5′DY782-labeled 26F DNA substrate and resolved on a urea-polyacrylamide denaturing gel. The non-incised substrate (S) and incision product (P) bands were visualized and quantified (left). Relative AP site incision efficiency. Shown are the the mean ± SD of three independent experiments (right). F, no immunoprecipitates. Developed by using the NIR Fluorescence technology.

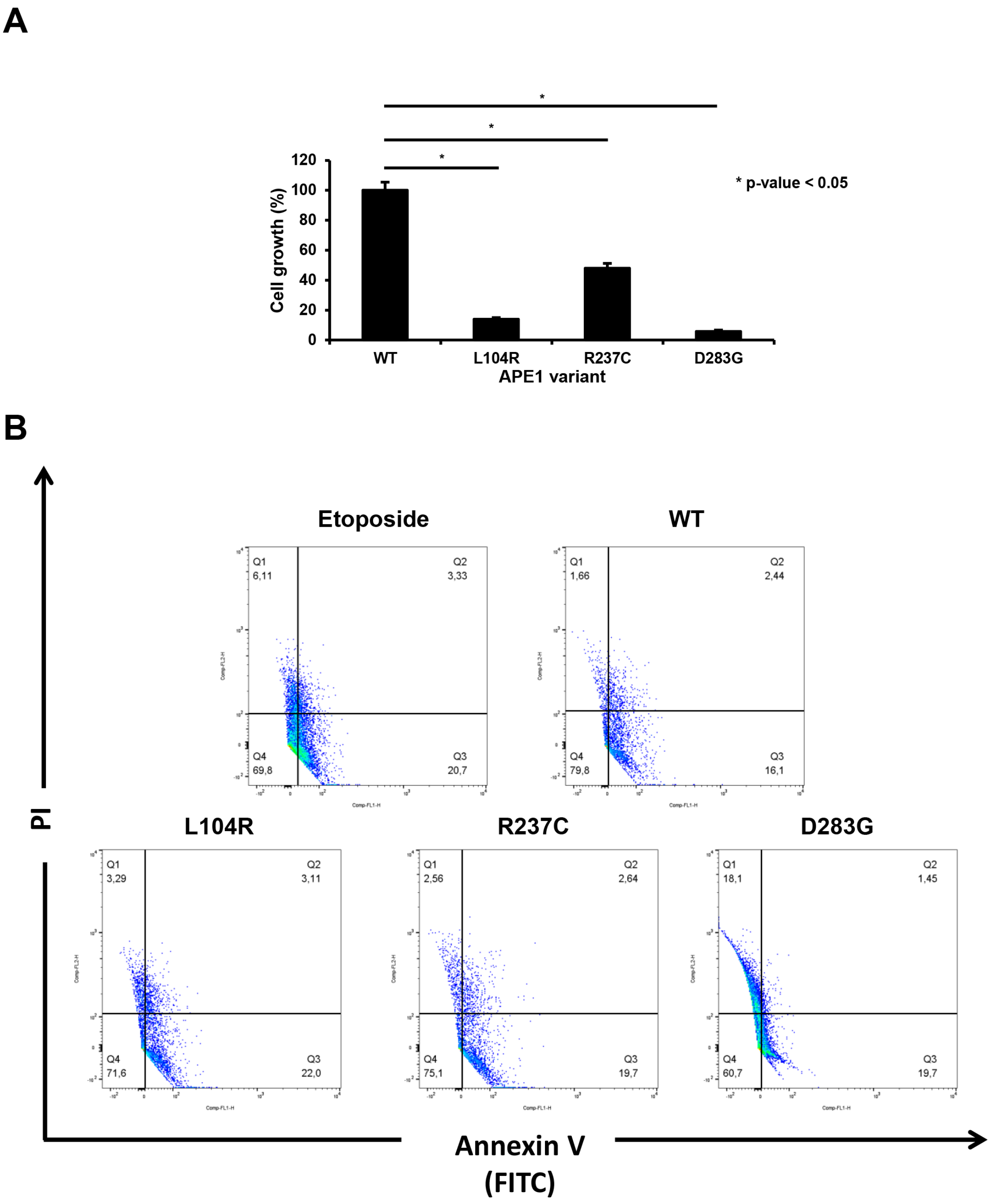

Supplementary Figure S5: APE1 variants show different growth capability. (**A**) Cell growth was measured by colony survival assay on APE1 reconstituted cell clones. Data are the mean ± SD of three independent experiments, considering WT as reference. (**B**) Flow cytometric analysis of early apoptotic and necrotic HeLa cell clones expressing APE1 genetic variants under basal conditions. Cells were harvested and double stained with annexin V-FITC (FL1-H) and PI (FL2-H); the early apoptotic cells (annexin V^+/PI^-) and necrotic (annexin V^+/PI^+) were analyzed by a dot-plot using a flow cytometer. Representative data are shown from three independent experiments. The percentage of cells that were annexin V positive, PI-positive or doubly positive for both annexin V and PI is indicated. As positive control for apoptosis, we used clones expressing $APE1^{WT}$ treated with 200 µM etoposide for 24 h (etop). X and Y axis are bioexponetially displayed.

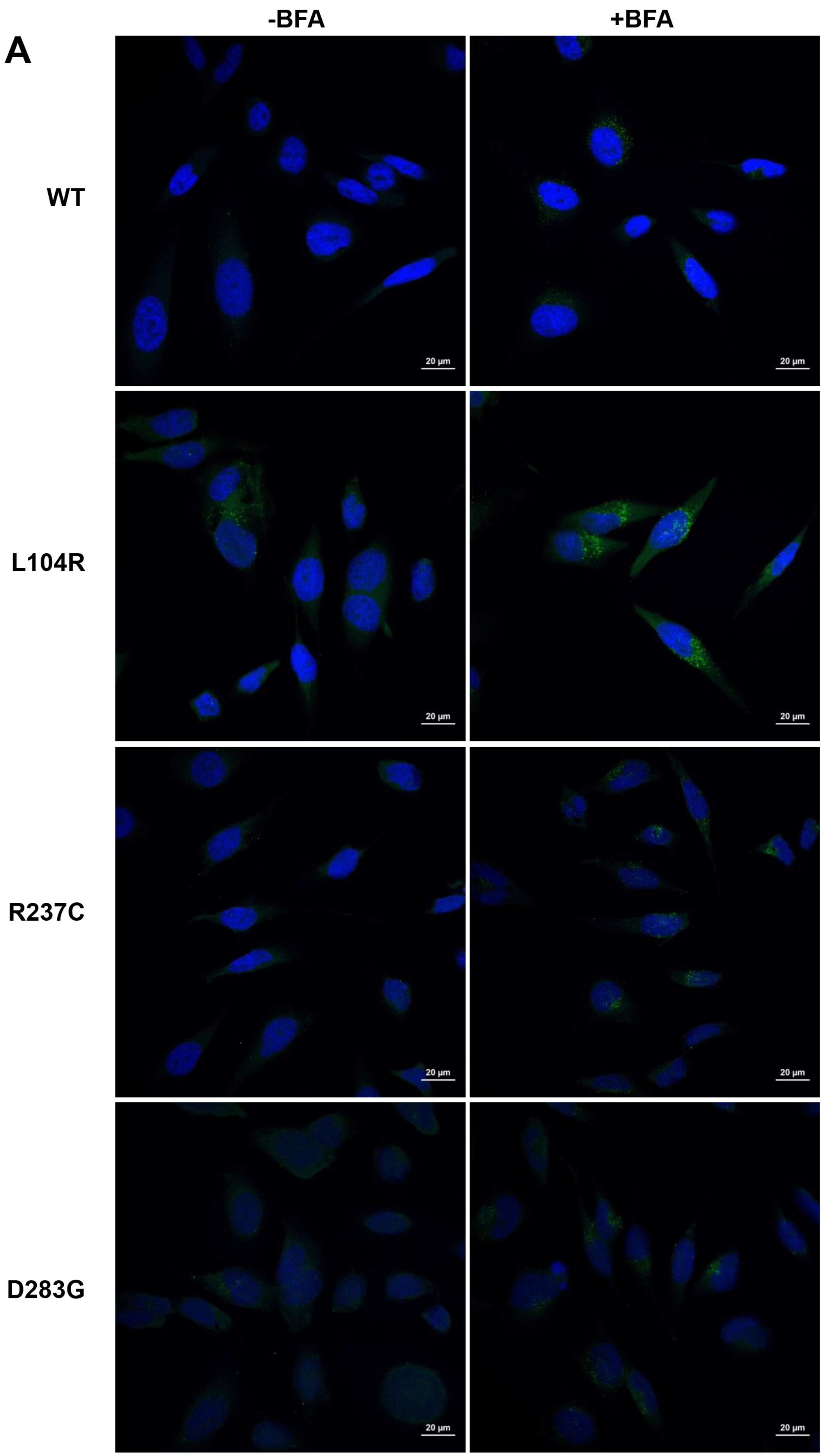
A
-BFA
+BFA
WT
L104R
R237C
D283G
20 µm

B

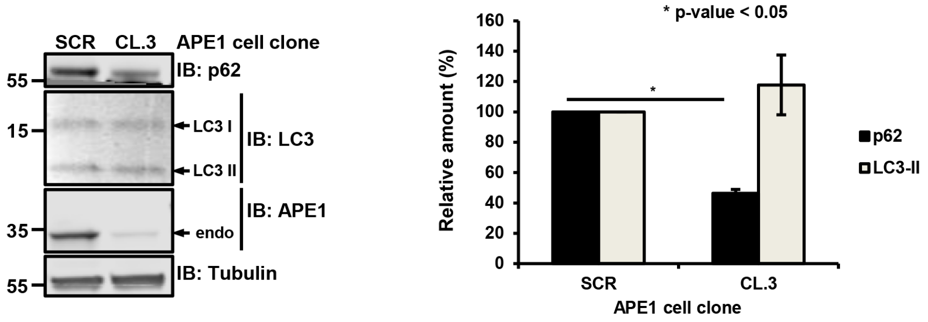

C

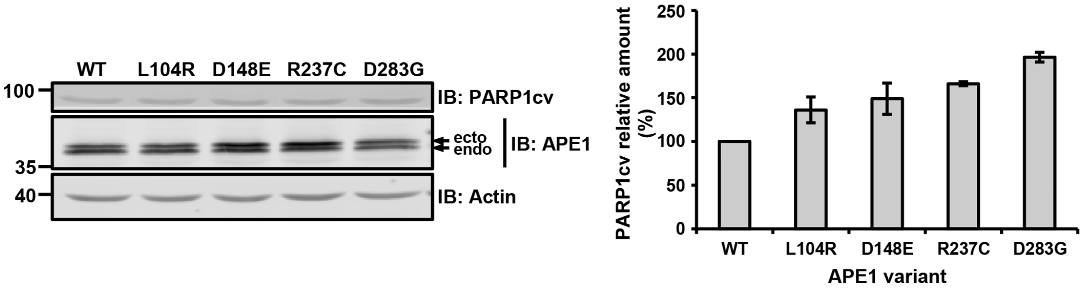

Supplementary Figure S6: Cellular senescence contribution to APE1 variants phenotype. (**A**) APE1 variants induce formation of LC3 puncta. Immunofluorescence showing increased accumulation of LC3 puncta in APE1 variants expressing clones. As positive control, cells were treated with bafilomycin A (100 nM for 4 h). (**B**) APE1 silencing determines an increased autophagy. Western blots showing autophagy induction in siAPE1 cell clone (CL.3) by monitoring p62 and LC3-II levels in whole cell extracts. Tubulin was used as loading control (top). Quantification of LC3-II and p62 levels, considering SCR as reference. LC3-II bands were normalized to those of tubulin (bottom). Developed by using the NIR Fluorescence technology. (**C**) APE1 polymorphisms show increased apoptotic PARP1 cleavage. Representative Western blots showing PARP1cv levels on transiently transfected HeLa cells. Actin was used as loading control (left). Quantification of PARP1cv levels, considering APE1^{WT} as a reference (right). Developed by using the NIR Fluorescence technology.

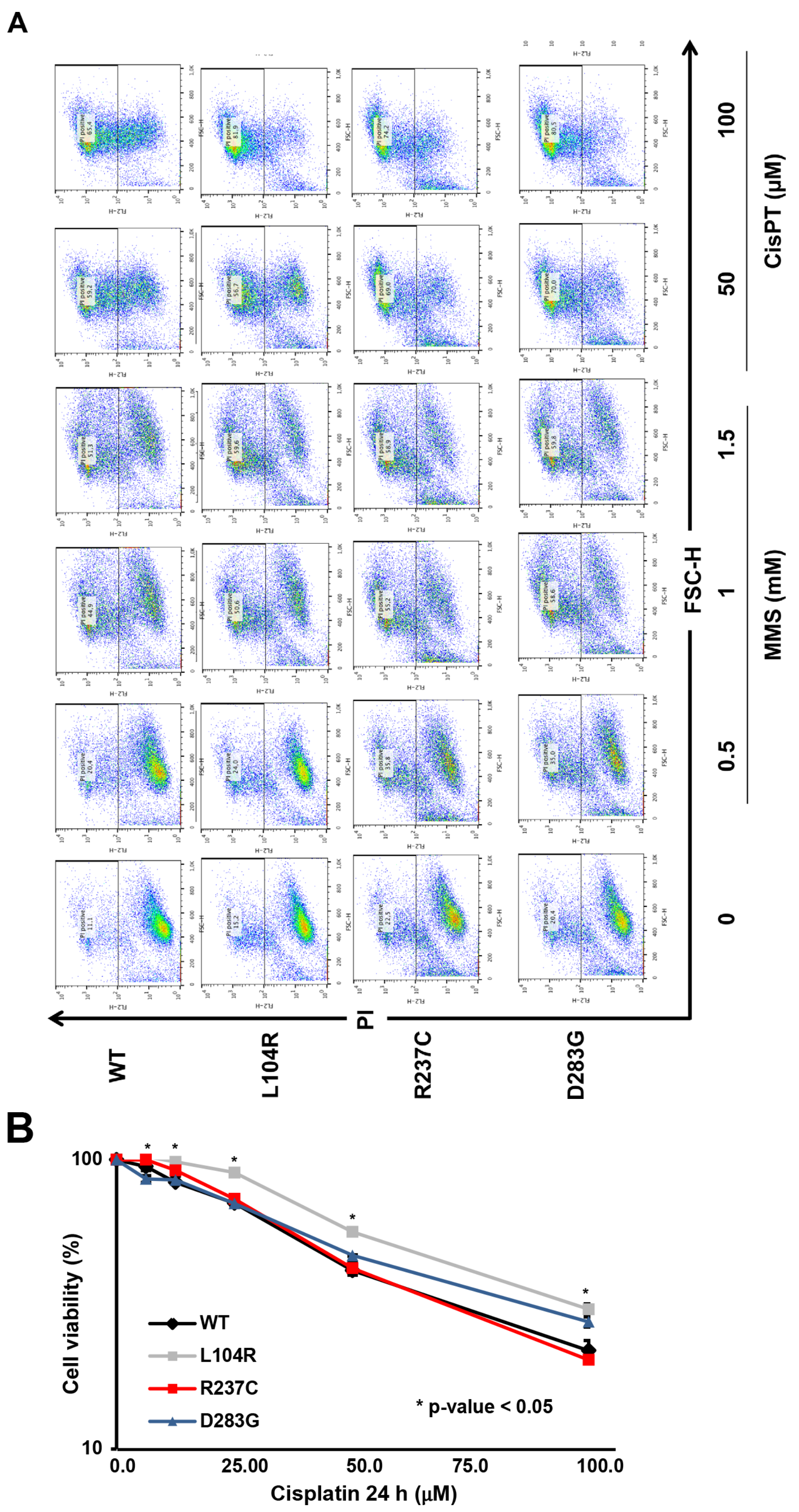

Supplementary Figure S7: Clones expressing APE1 genetic variants are less sensitive towards MMS and cisplatin. Panel A. Flow cytometric analysis of PI fluorescence to analyze cells apoptosis. HeLa cell clones expressing APE1 genetic variants were treated with different doses of MMS or cisplatin for 8 h or 24 h respectively. Cells were harvested and stained with PI. Representative data are shown from three independent experiments. FL2-H (PI) vs. FSC-H dot plot with gating on PI-positive. Panel B. Cell viability of APE1 variants-reconstituted clones subjected to treatment with increasing amounts of cisplatin was determined with a colorimetric (MTS) assay. Data expressed in logarithmic scale represent the mean ± SD of three independent experiments.

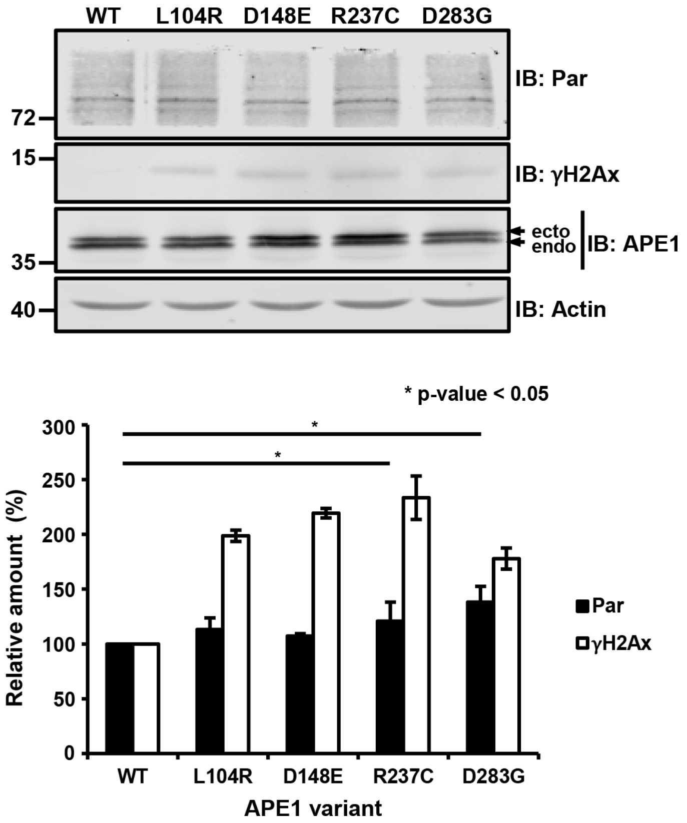

Supplementary Figure S8: APE1 polymorphisms lead to accumulation of PAR proteins and increased γH2Ax formation. Representative Western blots showing PAR protein and γH2Ax levels in transiently transfected HeLa cells. Actin was used as loading control (top). Quantification of PAR protein and γH2Ax levels in transiently transfected HeLa cells, considering $APE1^{WT}$ as a reference (bottom). Developed by using the NIR Fluorescence technology.
